# Clinical characteristics of endometrial cancers with microsatellite instability-high and *BRCA1/2* mutations

**DOI:** 10.3389/fonc.2025.1685642

**Published:** 2025-12-04

**Authors:** Yuping Shan, Yan Wang, Liying Huang, Guangqi Li, Zhumei Cui, Xiaoming Xing, Guangjie Yin

**Affiliations:** 1 Department of Gynecology, The Affiliated Hospital of Qingdao University, Qingdao, China; 2 Department of Pathology, The Affiliated Hospital of Qingdao University, Qingdao, China

**Keywords:** *BRCA1*, *BRCA2*, endometrial neoplasms, homologous recombination deficiency, microsatellite instability

## Abstract

**Objective:**

The purpose of this study was to identify the clinical features of endometrial cancers (EC) with microsatellite instability-high (MSI-H) and *BRCA1/2* mutations (*BRCA*m).

**Methods:**

We selected patients diagnosed with EC who were MSI-H from the Affiliated Hospital of Qingdao University between March 2021 and December 2024. The clinical information was collected after applying the inclusion and exclusion criteria. Based on *BRCA1/2* status, we divided the patients into two groups: the MSI-H only group (M group), and the MSI-H and *BRCA*m group (MB group). After comparing the clinical characteristics of the two groups, Kaplan-Meier (K-M) curves were employed to analyze the distribution of progression-free survival (PFS) between them. Finally, both univariate and multivariate Cox proportional hazards regression models were utilized to assess various prognostic variables.

**Results:**

A total of 177 patients were included in the final analysis, of whom 28 were identified as having *BRCA*m. The rate of laparoscopic surgery in M group was higher than that in MB group (87.25% *vs.* 67.86%, *p*-value=0.022). The lymph node metastasis rate in MB group was significantly higher than that in M group (14.29% *vs.* 2.01%, *p*-value=0.011). Additionally, a higher proportion of patients received immunotherapy in MB group (7.14% *vs.* 2.68%, *p*-value=0.011). The recurrence rates in M group and MB group were 1.34% and 17.86%, respectively (*p*-value=0.003). K-M analysis indicated that there was statistically significant difference in PFS between the two groups (*p*-value=0.006). Furthermore, we did not identify any independent risk factors that influenced prognosis in our study.

**Conclusion:**

The co-occurrence of *BRCA*m and MSI-H is associated with high rates of lymph node metastasis and recurrence. However, *BRCA*m was not an independent prognostic factor influencing PFS of EC with MSI-H.

## 1 Introduction

Endometrial cancer (EC) is the most prevalent gynecologic malignancy, with a significant rise in both incidence and mortality rates (1). Since the recent integration of genomic characterization into daily clinical practice by The Cancer Genome Atlas (TCGA), EC is classified into four categories (1): polymerase epsilon (*POLE*) ultramutated, characterized by *POLE* mutations (2); microsatellite instability hypermutated (MSI-H), identified by mismatch repair deficiency (MMRd) and high microsatellite instability (3); copy-number low (CN-L), which is defined by a lack of a specific molecular profile (NSMP); and (4) copy-number high (CN-H), primarily characterized by *TP53* mutations and serous-like EC (2, 3). MSI-H tumors exhibit a hypermutated phenotype and a high neoantigen load, making them particularly sensitive to immune checkpoint blockade (ICB) (4). Across various solid tumors, MSI-H status has emerged as a predictive biomarker for robust responses to PD-1/PD-L1 inhibitors (5). Recent phase II studies of neoadjuvant PD-1 blockade with dostarlimab in patients with MSI-H locally advanced rectal cancer have reported clinical complete responses in all treated individuals, allowing the omission of chemoradiotherapy and surgery in many cases and fundamentally challenging the traditional multimodal treatment paradigm (6). EC has the highest rate of MSI-H among all tumor types, with MSI-H present in 17% to 40% of EC cases (7, 8). Multiple clinical trials and real-world studies have demonstrated substantial and durable clinical benefits of ICB in patients with advanced, recurrent, or metastatic MSI-H EC (9, 10). These agents are now incorporated into major international guidelines as a standard systemic treatment option for this molecular subgroup. Therefore, MSI-H EC represents a biologically distinct and therapeutically actionable entity of significant clinical relevance. The primary cause of MSI-H is a defect in the DNA MMR genes responsible for correcting mismatched bases (7). Microsatellites are DNA segments composed of repeated sequences, typically ranging from one to six nucleotides in length (11). In cells with MMRd, these segments are susceptible to errors, leading to instability in their length and resulting in MSI-H (12). Particularly, hypermethylation of the mutL homolog 1 (*MLH1*) promoter (*MLH1*ph, 70%-75%), along with somatic mutations (15%-20%) and germline mutations (5%-10%) in *MLH1*, MutS homolog 2 (*MSH2*), *MSH6*, PMS1 homolog 2 (*PMS2*), and/or epithelial cell adhesion molecule (*EPCAM*), is associated with an accumulation of DNA replication errors in microsatellite regions (13, 14). Traditionally, MSI-H can be detected through immunohistochemistry (IHC) for MMR proteins absence, including MSH2, MSH6, MLH1, and PMS2, as well as through polymerase chain reaction (PCR)-based assays (11).

MSI-H EC typically exhibits a high tumor mutation burden (TMB-H), which is likely to predict a greater prevalence of homologous recombination (HR) gene variations (15, 16). HR is a multistep DNA repair process that addresses DNA double-stranded breaks (DSBs) (17). The *BRCA1* and *BRCA2* genes are the most altered HR genes and play crucial roles in the homologous recombination repair (HRR) pathway (18). Impaired function of the *BRCA1/2* can result in a deficiency in the HRR pathway, referred to as homologous recombination deficiency (HRD) (19). Previous reports have identified a potential co-occurrence of *BRCA1/2* mutations (*BRCA*m), which are targets for poly (ADP-ribose) polymerase inhibitors (PARPi), and MSI-H, a biomarker linked to responses to ICB (20–22). However, Sokol et al. found that since MSI-H tumors often contain thousands of mutations throughout the exome, some of these *BRCA*m may represent monoallelic bystander events that leave the second copy of *BRCA* intact, which does not lead to sensitivity to PARPi (22).

Emerging data indicate considerable heterogeneity within each molecular subgroup of EC. For example, within the NSMP category, specific patterns such as estrogen receptor (ER)-negative NSMP tumors have been associated with significantly worse outcomes, underscoring the need for further prognostic refinement within molecular classes (23). During routine review of molecular profiling reports at our institution, we observed that a subset of MSI-H EC harbored concomitant *BRCA*m. Given that MB are established biomarkers for responsiveness to ICB and PARPi, respectively, we hypothesized that MSI-H EC with co-occurring *BRCA*m might represent a distinct clinicopathologic subset with different treatment patterns and prognoses compared to MSI-H tumors without *BRCA*m. Therefore, we specifically focused on MSI-H EC and conducted an exploratory analysis of patients with or without *BRCA*m to generate preliminary data that may help refine prognostic assessment within this clinically important molecular subtype.

## 2 Materials and methods

This study was approved by the ethics committee of the Affiliated Hospital of Qingdao University (AHQU; approval number: QYFYWZLL 29592). Informed consent was acquired from the patients before the study started. All procedures performed in studies involving human participants were conducted in compliance with the ethical standards of the institutional and/or national research committee and in accordance with the 1964 Helsinki declaration and its subsequent amendments or equivalent ethical standards.

### 2.1 Study population selection

This retrospective study included patients diagnosed with EC who were MSI-H from AHQU between March 2021 and December 2024.

We provided information regarding genetic testing at AHQU below. (1) Genetic testing approach: Next-generation sequencing (NGS). (2) Reference genome: GRCh37/hg19. (3) Clinical database: 1000 Genomes, ExAC, gnomAD, ClinVar, BRCA Exchange InSiGHT, DBSNP, Cancer Hotspots, and COSMIC. (4) Mutation detection software: ADXHS-tBRCA-5p/ADXHS-gBRCA-CNV/ADXHS-tBPTM/ADXHS-gHRR-EN/ADXHS-tHRR-EN.

Patients were included in the final analysis if they met all of the following criteria: (1) diagnosis of EC confirmed by postoperative histopathology; (2) MSI-H status confirmed on NGS-based genetic testing and *BRCA*m status available; (3) initial treatment and primary surgical management for EC performed at AHQU; and (4) complete clinicopathologic, treatment, and follow-up data. Exclusion criteria were: (1) incomplete key clinical, pathological, treatment, or follow-up data; (2) patients who did not receive initial or surgical treatment for EC at AHQU; and (3) patients who were lost to follow-up.

### 2.2 Clinical characteristics

We divided the patients into two groups: the MSI-H only (M) and the MSI-H and *BRCA*m (MB) groups, based on their *BRCA1/2* status. The following variables were selected: age of diagnosis; body mass index (BMI); number of pregnancies; age at menopause; serum cancer antigen 125 (CA125) level before initial treatment; serum carbohydrate antigen 199 (CA199) level before initial treatment; serum carcinoembryonic antigen (CEA) level before initial treatment; surgical modality; perform pelvic lymph node dissection or not; postoperative pathology showed whether lymph node metastasis or not; histological type; histological grade; the International Federation of Gynecology and Obstetrics (FIGO) stage; postoperative immunohistochemical expressions of MMR proteins, ER, and progesterone receptor (PR); preoperative treatment; postoperative adjuvant treatment.

At our institution, pre-treatment serum CA125, CA199, and CEA are routinely measured in patients with suspected gynecologic malignancies as part of the standard preoperative evaluation. The purposes are to screen for possible concomitant gastrointestinal or ovarian malignancies when markedly elevated values are observed and to document baseline levels for subsequent surveillance in those patients with abnormal markers. In the present study, these tumor markers were recorded only as exploratory clinicopathologic variables and were not used to determine patient eligibility or to guide treatment decisions.

All patients were followed up every 2–4 months in the first 2 years after completing primary treatment. The follow-up interval was then extended to every 6–12 months in the subsequent 3 years. The follow-up period ended in October 2025. The monitoring protocol included a clinical and gynecologic examination at each visit; measurement of serum CA125, CA199, and CEA levels only in patients whose corresponding markers were elevated at baseline; and imaging studies when indicated by symptoms, physical findings, or abnormal tumor marker levels. Recurrent EC is defined as the recurrence of EC at any site after a patient has received initial treatment and has achieved a PFS of at least six months.

Overall survival (OS) and progression-free survival (PFS) were the primary study end points. OS was calculated from the date of diagnosis to the date of death from any cause, while PFS was calculated as the period from diagnosis to disease recurrence or progression.

### 2.3 Statistical analysis

The results were presented as means ± standard deviation (SD) for normally distributed variables, medians (25th percentile, 75th percentile) for non-normally distributed variables, numbers (percentages) for categorical variables. Univariate differences were analyzed using independent *t*-tests for normally distributed variables, Mann-Whitney *U* tests for non-normally distributed variables, and chi-square tests for categorical variables. Kaplan-Meier (K-M) curves were used to analyze the distribution of PFS across various groups. Furthermore, the log-rank test was applied to compare the differences between the curves. To evaluate various prognostic variables, univariate and multivariate Cox proportional hazards regression models were utilized to calculate hazard ratios (HR) and 95% confidence intervals (CI). To evaluate prognostic factors for PFS, we first performed univariate Cox proportional hazards regression analyses. Variables with a *p*-value < 0.05 in the univariate analysis were then included in the multivariable Cox model.

All statistical analyses were performed with SPSS software (version 29.0; IBM Corp., USA). A probability (*p*)-value of <0.05 was considered statistically significant.

## 3 Results

**Figure 1:**
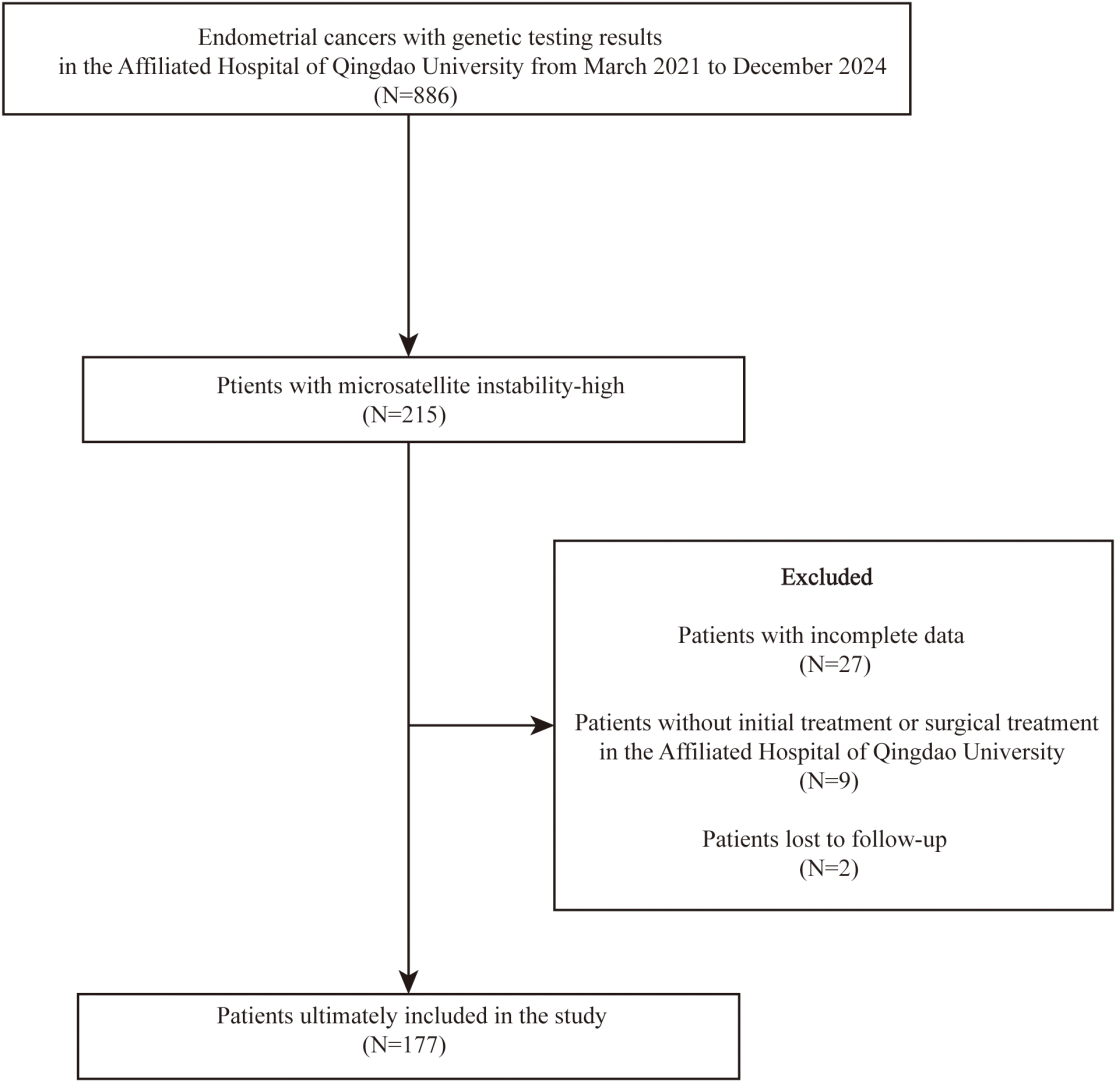
The flow diagram summarizing the selection process.

A total of 886 patients diagnosed with EC who had genetic testing results were identified at AHQU between March 2021 and December 2024. Among these, 215 patients were classified as MSI-H. Subsequently, 27 patients were excluded due to incomplete clinical data, 9 patients did not receive initial or surgical treatment at AHQU, and 2 patients were lost to follow-up. Ultimately, 177 patients were included in the final analysis (**Figure 1**).

### 3.1 Characteristics of the patients

**Table 1** showed the baseline characteristics of the selected patients. There were M and MB groups at 149 and 28 cases, respectively. In M group, 87.25% of patients underwent laparoscopic surgery, while 12.75% underwent open abdominal surgery. In MB group, 32.14% of patients underwent laparoscopic surgery, and 67.86% underwent open abdominal surgery. The difference between the two groups in the surgical modality was statistically significant (*p*-value=0.022). What’s more, the lymph node metastasis rate in MB group was significantly higher than that in M group (14.29% *vs.* 2.01%, *p*-value=0.011). In terms of postoperative adjuvant treatment, a larger number of patients in MB group received immunotherapy compared to those in M group. (7.14% (2/28) *vs.* 2.68% (4/149), *p*-value=0.011). Significantly, one patient underwent PARPi maintenance treatment following the completion of immunotherapy in our study. Additionally, the recurrence rate in MB group was 17.86%, while the recurrence rate in M group was 1.34%. This difference was statistically significant (*p*-value=0.003). No other significant differences were observed.

**Table 1:** Clinical characteristics of the patients in this study.

Characteristics	MSI-H only (n=149)	MSI-H and *BRCA*m (n=28)	*p*-value
Age of diagnosis (years)	58 (55, 64)	56 (54.75, 60)	0.392
BMI (kg/m²)	25.61 ± 3.33	25.42 ± 2.87	0.774
Number of pregnancies (times)	2 (2, 3)	3 (2, 3.25)	0.134
Age at menopause (years)	51 (49, 54)	51 (49.5, 53.25)	0.997
Serum CA125 level (U/ml)	19.77 (14.12, 31.71)	23.71 (13.86, 44.00)	0.203
Serum CA199 level (U/ml)	13.70 (9.01, 36.63)	20.94 (14.23, 94.19)	0.078
Serum CEA level (ng/ml)	1.72 (1.07, 2.73)	1.80 (1.57, 3.23)	0.073
Surgical modality			0.022
Open abdominal surgery	19 (12.75%)	9 (32.14%)	
Laparoscopic surgery	130 (87.25%)	19 (67.86%)	
Pelvic lymph node dissection			0.858
No	21 (14.09%)	3 (10.71%)	
Yes	128 (85.91%)	25 (89.29%)	
Lymph node metastasis			0.011
No	146 (97.99%)	24 (85.71%)	
Yes	3 (2.01%)	4 (14.29%)	
Histological type			0.707
Endometrioid carcinoma	143 (95.97%)	27 (96.43%)	
Clear cell carcinoma	1 (0.67%)	1 (3.57%)	
Serous carcinoma	2 (1.34%)	0 (0.00%)	
Mixed carcinoma	1 (0.67%)	0 (0.00%)	
Undifferentiated carcinoma	2 (1.34%)	0 (0.00%)	
Histological grade			0.980
Grade I	49 (32.89%)	9 (32.14%)	
Grade II	77 (51.68%)	15 (53.57%)	
Grade III	23 (15.44%)	4 (14.29%)	
FIGO stage			0.155
IA1	8 (5.37%)	1 (3.57%)	
IA2	99 (66.44%)	16 (57.14%)	
IA3	1 (0.67%)	0 (0.00%)	
IB	12 (8.05%)	1 (3.57%)	
IIA	10 (6.71%)	4 (14.29%)	
IIB	2 (1.34%)	0 (0.00%)	
IIC	1 (0.67%)	0 (0.00%)	
IIIA1	7 (4.70%)	1 (3.57%)	
IIIC1	4 (2.68%)	0 (0.00%)	
IIIC2	3 (2.01%)	2 (7.14%)	
IVA	0 (0.00%)	1 (3.57%)	
IVB	2 (1.34%)	1 (3.57%)	
IVC	0 (0.00%)	1 (3.57%)	
Loss of immunohistochemical expression of MMR proteins			0.323
MSH2+MSH6	17 (11.41%)	8 (28.57%)	
MLH1+PMS2	83 (55.70%)	13 (46.43%)	
MLH1+MSH6	1 (0.67%)	0 (0.00%)	
MLH1+MSH2	1 (0.67%)	0 (0.00%)	
PMS2	10 (6.71%)	0 (0.00%)	
MSH6	20 (13.42%)	5 (17.86%)	
MSH2	2 (1.34%)	0 (0.00%)	
No expression loss	15 (10.07%)	2 (7.14%)	
Immunohistochemical expression of ER			0.942
–	9 (6.04%)	1 (3.57%)	
+	140 (94.00%)	27 (96.43%)	
Immunohistochemical expression of PR			0.982
–	14 (9.40%)	2 (7.14%)	
+	135 (90.60%)	26 (92.86%)	
Preoperative treatment			0.221
No	144 (96.64%)	25 (89.29%)	
Chemotherapy	5 (3.36%)	3 (10.71)	
Postoperative adjuvant treatment			0.011
No	85 (57.05%)	12 (42.86%)	
Chemotherapy	28 (18.79%)	4 (14.29%)	
Immunotherapy	4 (2.68%)	2 (7.14%)	
Radiotherapy	12 (8.05%)	1 (3.57%)	
Chemotherapy+Immunotherapy	0 (0.00%)	3 (10.71%)	
Chemotherapy+Radiotherapy	18 (12.08%)	6 (21.43%)	
Chemotherapy+Immunotherapy+Radiotherapy	2 (1.34%)	0 (0.00%)	
Recurrence			0.003
No	147 (98.66%)	23 (82.14%)	
Yes	2 (1.34%)	5 (17.86%)	

(n=177). BMI, body mass index; *BRCA*m, *BRCA1/2* mutations; CA125, cancer antigen 125; CA199, carbohydrate antigen 199; CEA, carcinoembryonic antigen; ER, estrogen receptor; FIGO, the International Federation of Gynecology and Obstetrics; MMR, mismatch repair; MSI-H, microsatellite instability-high; MLH1, mutL homolog 1; MSH2, MutS homolog 2; MSH6, MutS homolog 6; PMS2, PMS1 homolog 2; PR, progesterone receptor.

### 3.2 Details of *BRCA*m

**Table 2** showed the detail of *BRCA*m sites in this study. Four patients had a *BRCA1* mutation, 19 patients had a *BRCA2* mutation, and five patients had both *BRCA1* and *BRCA2* mutation. Most patients in this study had an exon 11 mutation. Additionally, the c.1961 deletion and the c.9097 deletion were the most common nucleotide changes in *BRCA1* mutation group and *BRCA2* mutation group, respectively.

**Table 2:** The summary of *BRCA*m sites in this study.

Patients	Genes	Mutation location	Nucleotide change	Mutation abundance
1	*BRCA1*	exon11	c.1961 deletion	11.13%
2	*BRCA1*	exon11	c.1961 deletion	11.13%
3	*BRCA1*	exon11	c.1961 deletion	10.35%
4	*BRCA1*	exon11	c.1961 deletion	3.45%
5	*BRCA2*	exon5	c.475 G>T	27.72%
6	*BRCA2*	exon10	c.1038 duplication	21.29%
7	*BRCA2*	exon11	c.2175 duplication	5.97%
8	*BRCA2*	exon11	c.2588 deletion	10.02%
9	*BRCA2*	exon11	c.2957 deletion	5.96%
10	*BRCA2*	exon11	c.3248 deletion	20.03%
11	*BRCA2*	exon11	c.5164_5165 deletion	50.00%
12	*BRCA2*	exon11	c.5281 G>T	3.00%
13	*BRCA2*	exon11	c.5351 duplication	3.02%
14	*BRCA2*	exon11	c.5351 duplication	35.02%
15	*BRCA2*	exon17	c.7886 G>A	5.57%
16	*BRCA2*	exon17	c.7971 deletion	13.37%
17	*BRCA2*	exon19	c.8420 C>A	9.85%
18	*BRCA2*	exon23	c.9097 deletion	41.75%
19	*BRCA2*	exon23	c.9097 deletion	13.30%
20	*BRCA2*	exon23	c.9097 deletion	12.15%
21	*BRCA2*	exon23	c.9097 duplication	12.10%
22	*BRCA2*	exon23	c.9097 duplication	7.96%
23	*BRCA2*	exon23	c.9253 deletion	15.94%
24	*BRCA1*	exon11	c.1080 C>A	4.88%
	*BRCA2*	exon11	c.2957 duplication	15.15%
25	*BRCA1*	exon11	c.1961 deletion	4.02%
	*BRCA2*	exon11	c.2957 deletion	3.24%
26	*BRCA1*	exon11	c.3108 deletion	15.45%
	*BRCA2*	exon23	c.9097 deletion	26.32%
27	*BRCA1*	exon11	c.1016 deletion	5.47%
	*BRCA2*	exon18	c.8009 C>T	3.76%
	*BRCA2*	exon24	c.9253 deletion	9.95%
28	*BRCA2*	exon3	c.100 G>T	25.46%
	*BRCA2*	exon10	c.1650 G>T	22.96%
	*BRCA2*	exon11	c.3344 C>A	25.20%
	*BRCA2*	exon11	c.4321 G>T	24.75%
	*BRCA2*	exon14	c.7297 C>T	29.33%
	*BRCA2*	intron3	c.317–1 G>T	26.43%
	*BRCA2*	intron9	c.794–1 G>T	29.94%

*BRCA*m, *BRCA1/2* mutations.

### 3.3 Survival analysis and prognostic factors

**Figure 2:**
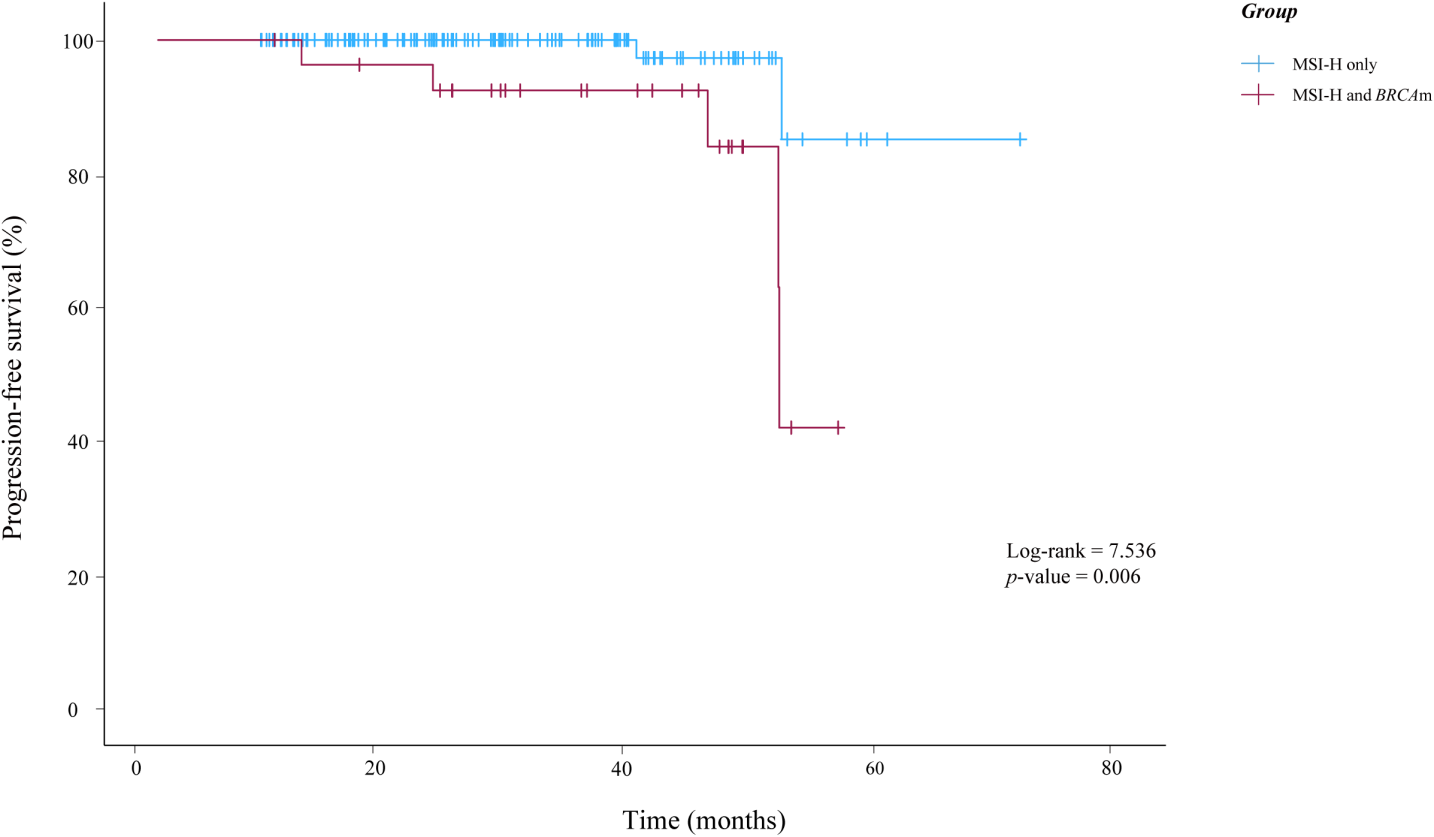
Kaplan-Meier curves illustrating the progression-free survival of patients in this study. *BRCA*m, *BRCA1/2* mutations; MSI-H, microsatellite instability-high.

In this study, only seven patients experienced a relapse. Of these, two patients presented with MSI-H alone, while five patients exhibited both MSI-H and *BRCA*m. Notably, no deaths have been reported as of October 2025 in this study. According to the K-M survival analysis presented in **Figure 2**, there was statistically significant difference in PFS between the two groups (*p*-value=0.006).

In the univariate analysis using the Cox proportional hazards regression model, significant prognostic factors included serum CA125 level (*p*-value=0.004), serum CA199 level (*p*-value=0.049), lymph node metastasis (*p*-value=0.012), preoperative treatment (*p*-value=0.034), and *BRCA*m (*p*-value=0.014). These five variables were therefore included in the multivariable Cox model. In the multivariable analysis adjusting simultaneously for these covariates, none of them remained statistically significant (all *p*-value > 0.05). The details of the univariate and multivariate Cox regression analyses were presented in **Table 3**.

**Table 3:** Univariate and multivariable Cox proportional hazard regression analysis of progression-free survival.

Characteristics	Univariate analysis		Multivariate analysis	
	Hazard ratios (95% CI)	*p*-value	Hazard ratios (95% CI)	*p*-value
Age of diagnosis (years)	0.329 (0.799, 1.245)	0.300		
BMI (kg/m²)	0.655 (0.512, 1.231)	0.241		
Number of pregnancies (times)	0.900 (0.513, 1.719)	0.754		
Age at menopause (years)	0.799 (0.576, 1.189)	0.227		
Serum CA125 level(U/ml)	1.015 (1.003, 1.211)	0.004	1.235 (0.991, 1.566)	0.572
Serum CA199 level(U/ml)	1.123 (1.000, 1.009)	0.049	0.998 (0.873, 2.235)	0.792
Serum CEA level(ng/ml)	1.130 (0.929, 1.224)	0.893		
Surgical modality		0.078		
Open abdominal surgery	Reference			
Laparoscopic surgery	0.234 (0.144, 1.385)			
Pelvic lymph node dissection		0.200		
No	Reference			
Yes	0.378 (0.101, 1.795)			
Lymph node metastasis		0.012		0.889
No	Reference		Reference	
Yes	14.200 (2.013, 88.102)		0.452 (0.000, 20535.104)	
Histological type		0.999		
Endometrioid carcinoma	Reference			
Clear cell carcinoma	0.052 (0.000, 9.212E + 24)			
Serous carcinoma	0.045 (0.000, 2.113E + 12)			
Mixed carcinoma	0.043 (0.000, 8.058E + 13)			
Undifferentiated carcinoma	0.045 (0.000, 4.114E + 12)			
Histological grade		0.987		
Grade I	Reference			
Grade II	1.236 (0.200, 7.936)			
Grade III	0.000 (0.000, -)			
FIGO stage		0.078		
IA1	Reference			
IA2	312.470 (0.000, 2.111E + 52)			
IA3	0.000 (0.000, -)			
IB	0.531 (0.000, 3.199E + 77)			
IIA	6.514 (0.000, 3.423E + 51)			
IIB	–			
IIC	–			
IIIA1	5.724 (0.000, 1.351E + 53)			
IIIC1	5.910 (0.000, 3.567E + 55)			
IIIC2	4.232 (0.000, 2.272E + 55)			
IVA	2502.518 (0.000, 1.694E + 53)			
IVB	6.577 (0.000, 3.234E + 55)			
IVC	30004.285 (0.000, 2.007E + 54)			
Loss of immunohistochemical expression of MMR proteins		0.999		
MSH2+MSH6	Reference			
MLH1+PMS2	0.634 (0.098, 3.632)			
MLH1+MSH6	0.000 (0.000, -)			
MLH1+MSH2	0.000 (0.000, -)			
PMS2	0.000 (0.000, -)			
MSH6	0.000 (0.000, -)			
MSH2	0.000 (0.000, -)			
No expression loss	0.000 (0.000, -)			
Immunohistochemical expression of ER		0.688		
–	Reference			
+	23.440 (0.000, 17198098.284)			
Immunohistochemical expression of PR		0.672		
–	Reference			
+	32.310 (0.000, 5182056.768)			
Preoperative treatment		0.034		0.900
No	Reference		Reference	
Chemotherapy	9.323 (1.477, 52.523)		0.444 (0.000, 26367.920)	
Primary treatment after surgery		0.345		
No	Reference			
Chemotherapy	0.000 (0.000, -)			
Immunotherapy	22.375 (1.207, 379.273)			
Radiotherapy	0.000 (0.000, -)			
Chemotherapy+Immunotherapy	25.789 (2.189, 290.808)			
Chemotherapy+Radiotherapy	4.929 (0.283, 90.900)			
Chemotherapy+Immunotherapy+Radiotherapy	0.000 (0.000, -)			
*BRCA*m		0.014		0.056
–	Reference		Reference	
+	34.728 (1.343, 108.979)		8.020 (0.307, 92.343)	

BMI, body mass index; *BRCA*m, *BRCA1/2* mutations; CA125, cancer antigen 125; CA199, carbohydrate antigen 199; CEA, carcinoembryonic antigen; ER, estrogen receptor; FIGO, the International Federation of Gynecology and Obstetrics; MMR, mismatch repair; MSI-H, microsatellite instability-high; MLH1, mutL homolog 1; MSH2, MutS homolog 2; MSH6, MutS homolog 6; PMS2, PMS1 homolog 2; PR, progesterone receptor.

## 4 Discussion

Currently, the number of targeted cancer therapies based on genomic profiles has increased significantly, contributing to the advancement of personalized medicine (22). However, when genomic profiling reveals multiple potential therapeutic options, it creates a diagnostic dilemma in which the optimal initial choice of systemic therapy may be uncertain. Moore et al. reported a case of progressive metastatic prostate cancer characterized by the co-occurrence of MSI-H and *BRCA*m (24). They suggested that the ICB should be considered before PARPi for these patients because *BRCA*m are likely carriers of MSI-H but are unlikely to be driver molecular alterations (24). As the tumor type with the highest rate of MSI-H, we investigated whether EC exhibiting both MSI-H and *BRCA*m displays distinct clinical characteristics. We explored the clinical features, treatment patterns, and outcomes associated with MSI-H and *BRCA*m in EC.

Our genetic testing data is obtained through NGS, which provides high sensitivity and comprehensive detection capabilities (25). In this study, the molecular typing rates of EC that we observed were comparable to those reported in other studies. And we found that the median ages in these two groups were 58 and 56 years, which is younger than the average age at diagnosis of 63 years (26). The data from the Surveillance, Epidemiology, and End Results (SEER) program, which spans from 1990 to the present, indicate a consistent increase in cases among women under the age of 50. The average BMI was approximately 25 kg/m², which is classified as overweight in our study. Compared to other cancers, EC has the strongest association with obesity (27). A recent meta-analysis of 30 prospective studies found that each 5 kg/m² increase in BMI was associated with a 54% higher risk of developing EC (28). A national cohort study in Denmark demonstrated that the risk of EC is reduced, regardless of whether a pregnancy ends shortly after conception or at 40 weeks of gestation, due to biological processes occurring within the first weeks of pregnancy (29). Our study found that the median number of pregnancies was 2 and 3, respectively. The average age at menopause in our study was approximately 51 years. A recent study reported a positive association between the age at menopause and EC suggesting that late menopause increases the risk of developing EC (30). We found that the median levels of serum CA125, CA199, and CEA were normal in this study. Similarly, previous literature has reported on the role of various serum tumor markers in EC, indicating that elevations occur in only 20% to 30% of patients (31). Lymph node assessment is critically important to decide treatment in EC and most patients in our study received pelvic lymph node dissection. However, lymph node dissection carries specific surgical risks and potential complications, and in response, a sentinel lymph node strategy has been developed and refined over the past decade (32). Moreover, the majority of histological grades were classified as grade II, and the FIGO stage was IA2. This indicates that most patients with EC were in the early stages and had moderately differentiated tumors. This finding aligns with the fact that nearly 75% of patients with EC present with FIGO stage I disease (26). Particularly, the proportion of patients with stage IV was higher in MB group compared to M group, however, this difference was not statistically significant due to the small number of cases. In our study, MB group did not exhibit distinct immunohistochemical characteristics. Immunohistochemistry for MMR proteins, including MLH1, MSH2, MSH6, and PMS2, has emerged as the preferred diagnostic surrogate for MSI globally (33). MSI-H typically indicates reduced or deficient activity of the MutSα protein complex, which is responsible for the initial recognition of DNA mismatches. This reduction can occur due to mutations or inactivation of the heterodimer partners MSH2 or MSH6. Additionally, the MutLα protein complex, which subsequently nicks DNA at sites of mismatch to initiate repair, may also exhibit reduced activity due to mutations or inactivation of its heterodimer partners MLH1 or PMS2. The loss of MLH1/PMS2 or MSH2/MSH6 expression is the most commonly observed pattern in MSI-H tumors (34). Moreover, a significant proportion of all EC cases express both ER and PR (34). The immunohistochemical expressions of MMR proteins, ER, and PR in our study were consistent with previous study. Multiple prospective studies have aimed to identify women with early-stage disease who are at the highest risk of relapse and to develop effective adjuvant therapies (26). However, to date, no such strategy has demonstrated an improvement in OS. And most patients in our study did not receive neoadjuvant therapies. In our study, the clinical characteristics of MB group mentioned above did not appear to be unique.

In this study, we found that the number of patients who underwent laparoscopic surgery was higher than that of those who had open abdominal surgery. Since the adoption of laparoscopic surgery for EC, the incidence of laparoscopic procedures has gradually increased compared to open surgery. This is attributed to the fact that laparoscopic surgery offers oncologic outcomes comparable to those of open surgery, while also reducing surgical morbidity and improving the quality of life for patients (35). Laparoscopic surgery has become the standard approach in modern medical practice, offering significant advantages. In our study, the proportion of open abdominal surgeries in MB group was higher than that in M group. This discrepancy may be attributed to the fact that patients in the former group had a greater likelihood of extensive metastasis to lymph nodes and other sites, which influenced the choice of surgical methods. We interpret the observed association between MB group and a higher rate of open surgery as an indirect indication of a greater disease burden in some patients. In our study, patients in MB group more frequently exhibited lymph node metastasis and showed a numerically higher proportion of advanced FIGO stage. However, this latter difference did not reach statistical significance, likely due to the limited sample size. These factors may have influenced surgeons to prefer laparotomy over laparoscopy in selected cases to ensure thorough exploration and cytoreduction. Additionally, MB group exhibited higher rates of lymph node metastasis and recurrence. Generally, MSI-H and *BRCA*m typically exhibit a TMB-H and increased chromosomal fragmentation compared to MSI-H alone, which may indicate a higher likelihood of lymph node metastasis and recurrence. Preoperative treatment is reserved for patients with more advanced tumor stages or unfavorable presentations, such as bulky pelvic or para-aortic lymph nodes, suspected distant metastases, or poor general condition that makes immediate standard surgery unsafe. Therefore, receiving preoperative treatment primarily reflects a worse baseline disease status rather than the direct effect of a specific molecular subtype. Since MSI-H and *BRCA*m status were determined from postoperative NGS, these molecular features did not influence the decision to administer neoadjuvant therapy. Consequently, the association between preoperative treatment and prognosis observed in the univariate analysis should be interpreted as an indicator of more aggressive disease rather than evidence that preoperative therapy itself worsens outcomes in MSI-H EC. Specifically, MSI-H typically induces a robust anti-tumor immune response, characterized by the infiltration of CD8+ T cells (36). However, *BRCA*m not only upregulate the expression of Snail and vascular endothelial growth factor (VEGF), promoting epithelial-mesenchymal transition (EMT) and angiogenesis, but also recruit immunosuppressive cells, such as myeloid-derived suppressor cells (MDSCs), which can facilitate immune evasion by tumors (37). The 2023 National Comprehensive Cancer Network (NCCN) Guidelines for Uterine Tumors (Version 2) recommend immunotherapy as a first-line treatment for advanced (stage III-IV) and recurrent metastatic EC for the first time, based on findings from the NRG-GY018 trial and the RUBY trial (38). The use of immunotherapy in patients with MSI-H and/or *BRCA*m in our cohort aligns with current guideline-based practices and is not intended to be presented as a novel therapeutic strategy. The proportion of patients receiving immunotherapy was higher in MB group compared to M group. In MB group, five patients received either immunotherapy or chemotherapy in combination with immunotherapy, of which three patients experienced a recurrence (recurrence time: 4, 17, and 30 months). However, there was no recurrence among the patients who received either immunotherapy or chemotherapy in combination with immunotherapy in M group. This may be related to our above discussion that patients with MSI-H and *BRCA*m may have a higher TMB. Consequently, these patients are more likely to experience relapse or metastasis, leading to a higher proportion receiving immunotherapy. In our study, some advanced patients did not receive immunotherapy either because they were diagnosed with EC before 2023 or due to financial constraints. Patients in MB group exhibited a higher likelihood of recurrence, despite receiving a greater proportion of postoperative adjuvant treatment. Those who received immunotherapy, whether in combination with chemotherapy or not, were selected for individual analysis, and patients in the *BRCA*m group also demonstrated a higher recurrence rate.

According to previous literature, 83% of MSI-H patients are classified as TMB-H, while both MSI-H and TMB-H/microsatellite stable (MSS) patients show a higher rate of *BRCA*m (22). This mutation is a single allelic mutation and is categorized as a bystander effect. It does not lead to abnormal poly (ADP-ribose) polymerase function, indicating that there will be no positive treatment response to PARPi. We speculate that, compared to patients with MSI-H only, those with MSI-H and *BRCA*m exhibit a higher TMB, which may contribute to increased tumor metastasis and recurrence. Based on this inference, we do not routinely administer PARPi to EC with MSI-H and *BRCA*m to avoid exposing them to potential side effects without a corresponding likelihood of therapeutic benefit. In our study, one patient chose to undergo PARPi maintenance treatment following the completion of immunotherapy, and no disease recurrence has been observed in this individual. Further research and clinical observations are necessary to determine whether patients with MSI-H and *BRCA*m indeed have a higher TMB, whether this is a bystander effect, and whether such patients can derive benefits from PARPi treatment.

Exon11 was the most common mutation location in our study. Similarly, among *BRCA*m in ovarian cancer, exon11 is also the most common mutation location. This can be attributed to the fact that exon 11 of *BRCA1/2* is particularly large, with approximately half of the mutations occurring in this region (39). We observed differences in PFS between the two groups, with patients carrying *BRCA*m exhibiting a shorter PFS. But we did not identify any independent risk factors that influenced prognosis in our study. However, some studies have identified independent risk factors that affect the prognosis of patients with EC. For example, the PORTEC-3 trial demonstrated that patients with stage III disease who received chemoradiation therapy followed by four cycles of carboplatin and paclitaxel chemotherapy experienced improved OS and five-year PFS rates compared to those who underwent radiation therapy alone (40, 41). Additionally, age, lymph node metastasis, histological type, histological grade, FIGO stage, and other factors are also considered to be related to the patient’s prognosis (26, 42, 43).

Some limitations also need to be considered in this study. First, because our cohort included patients treated up to 2024, the follow-up period for the most recently treated patients was relatively short, which may lead to underestimation of late recurrences and survival events. Therefore, our findings should be interpreted with caution, and longer-term follow-up and external validation are warranted. Second, a small number of patients with MSI-H EC were lost to follow-up (2/215, 0.9%) or excluded due to incomplete baseline data. If these patients had systematically worse outcomes than those who remained in the cohort, this could have introduced attrition bias and non-random missingness, potentially leading to an overestimation of PFS. Because outcome data were unavailable for these individuals, we could not fully rule out this possibility. Third, this study involved a surgery-based cohort. We included only patients who underwent primary surgical treatment at our institution and had complete clinical data. Patients with inoperable disease, poor performance status, or incomplete records were excluded from our analysis, which may introduce potential selection bias. Finally, this was a retrospective, single-center study that included only 28 patients with concurrent *BRCA*m. We hope our study will encourage future multi-center research with larger cohorts and longer follow-up periods to validate our findings and more reliably identify independent prognostic factors.

## 5 Conclusion

Compared to M group, MB group showed a higher rate of lymph node metastasis, immunotherapy, and recurrence. Although the K-M curves indicated that patients with *BRCA*m experienced shorter PFS compared to patients with MSI-H only, *BRCA*m was not identified as an independent prognostic factor influencing PFS in patients with MSI-H. In summary, the coexistence of MSI-H and *BRCA*m may indicate a higher TMB, which is associated with increased rates of lymph node metastasis and recurrence compared to M group. We hope that this single-center study will inspire further research that includes longer follow-up periods and a larger patient population. This will provide a more comprehensive understanding of the clinical characteristics of EC with MSI-H and *BRCA*m, thereby facilitating the selection of more precise and personalized treatment strategies.

## Data Availability

The original contributions presented in the study are included in the article/supplementary material. Further inquiries can be directed to the corresponding authors.
